# Historically Accurate Reconstruction of the Materials and Conservation Technologies Used on the Facades of the Artistic Buildings in Lecce (Apulia, Italy)

**DOI:** 10.3390/ma15103658

**Published:** 2022-05-20

**Authors:** Daniela Fico, Daniela Rizzo, Raffaele Casciaro, Carola Esposito Corcione

**Affiliations:** 1 Dipartimento di Ingegneria dell’Innovazione, Università del Salento, Edificio P, Campus Ecotekne, s.p. 6 Lecce-Monteroni, 73100 Lecce, Italy; daniela.fico@unisalento.it (D.F.); carola.corcione@unisalento.it (C.E.C.); 2 Dipartimento di Beni Culturali, Università del Salento, Via D. Birago 64, 73100 Lecce, Italy; raffaele.casciaro@unisalento.it

**Keywords:** stone, buildings, protective coatings, FTIR spectroscopy, pyrolysis–gas chromatography–mass spectrometry

## Abstract

The protection of the stone surfaces of the buildings of the city of Lecce (Apulia, Italy) represents an ancient practice, which has always allowed the conservation of the historical-artistic heritage of the city, which nowadays is an international touristic and cultural destination. The identification of ancient recipes, materials and methodologies for the protection of historical buildings plays an important role in establishing correct protocols in order to ensure the durability of stone surfaces over time. This work presents a historically accurate reconstruction of the materials and conservation technologies used on the facades of the artistic buildings in Lecce. Several historical buildings, both civil and religious, have been selected in order to investigate the treatments applied on their facades and to know the traditions spread in the past in the field of building conservation in the Salento territory. Thanks to non-invasive or micro-destructive techniques (optical microscopy, ATR-FTIR spectroscopy, pyrolysis–gas chromatography–mass spectrometry), the characteristic molecular markers of the materials and the products of degradation have been identified, deepening the knowledge of the mechanisms of deterioration and interaction between the stone material, the surface finish and the surrounding environment. The paper is a valuable tool for the knowledge of ancient traditions and the planning of proper restoration works.

## 1. Introduction

The city of Lecce (Apulia, southern Italy) is famous for its architecture, which has developed mostly between the 17th and 19th centuries [1,2,3,4]. Dolmens, sculptures, statues, buildings and decorations have been built from prehistoric times to the more famous Baroque period, thanks to the use of two local stone materials, “pietra leccese” and “carparo”, both biocalcarenites with golden colors that are easy to carve [3,5,6]. However, these stone materials have a low durability and are susceptible to degradation in the external environment due to their porosity and low degree of cementation [7,8,9]. In fact, among the different varieties of Lecce stone, most of the historical buildings in the city of Lecce were built with the Cursi variety, which has a percentage of porosity between 30 and 44% [7,8]. The main causes of deterioration are physical, chemical and biological. Among these, the most evident phenomena of deterioration are the crystallization of soluble salts; the formation of efflorescence, subflorescence, black crusts and patinas; alveolization; biological alteration; and others [3,7].

These causes led to the need to intervene on stone materials through conservation practices with the aim of slowing down or inhibiting the deterioration process through the use of biocidal, consolidating or water-repellent treatments [10,11,12,13,14]. Among these, the application of water-repellent treatments on the facades of buildings was most widely used in the Lecce area, between the 17th and 19th centuries [3,6,15,16,17]. However, the original documents relating to their use are almost non-existent in the historical archives, and there are only few manuscripts that report the water-repellent materials and the ancient methodologies used by the craftsmen. Furthermore, these manuscripts are often generic, and it is not possible to establish any correlation between the maintenance practice and the state of conservation of the buildings [3,10]. In general, manuscripts and traditions handed down orally over time refer to organic and inorganic materials used on the facades of the stone buildings. These substances were used on their own or as binders to produce colored or transparent water-repellent surfaces. These surface treatments are often very difficult to identify correctly, due to the reduced thickness and to the presence of multiple materials, often not yet chemically characterized [10]. Calia et al. [15] carried out optical microscopy, SEM-EDS, FTIR and XRD analysis on samples taken from the facades of buildings of historical-artistic value in Lecce (the churches of *Santa Elisabetta* and the *Santi Niccolò and Cataldo*) and in Melpignano (*San Giorgio Church*). The authors determined the presence and distribution of clay minerals within the surface layers of the investigated monuments and established their different origins, showing that, in some cases, they were intentionally used in the facades of the buildings for aesthetic and conservation purposes [15].

Our recent research has concerned the so-called “technical art history” [10]. Thanks to the reproduction of ancient preservation recipes applied to stone specimens (“pietra leccese” and “carparo”) and the use of various analytical techniques, biomarkers characteristic of the various natural water-repellent substances used in the past were identified [10]. A complete chemical characterization has been carried out, even for what regards natural water-repellents for stone materials, such as the extract of the plant species *Opuntia Ficus-Indica* and *Drimia maritima*, not previously investigated in the field of cultural heritage. Moreover, natural and accelerated aging tests have provided information on their chemical decomposition processes and degradation products [10]. The results obtained from this study were used to investigate the chemical composition of samples taken from the facades of three historical buildings located in Lecce and its province [18]: the *Church of San Giorgio* (Melpignano, Lecce) and the *Church of SS. Niccolò and Cataldo* (Lecce) [18], previously and partially studied in the work of Calia et al. [15], and *Palazzo Maggio* (Corigliano d’Otranto, Lecce). In this work, the study of surface finishes intentionally applied for conservation purposes has been deepened, investigating the chemical composition of the organic mixtures used in the past. Through the use of pyrolysis–gas chromatography–mass spectrometry (Py-GC/MS), with and without hydrolysis and thermally assisted methylation, the presence of ancient surface treatments, previously only hypothesized by written sources, has been ascertained [18]. Moreover, for the first time, the use of *Opuntia Ficus-Indica* leaf as a natural protective treatment of Lecce stone has been scientifically confirmed, thanks to the identification of some characteristic chemical biomarkers [18].

In spite of the progress made in recent years on the knowledge of the ancient methodologies and practices of conservation of buildings used in the territory of Lecce, the case studies analyzed are still few and lack a wider survey, which would also allow discriminating between treatments carried out on religious or civil buildings. The craftsmen and the workshops to which the clients turned might be different according to the importance of the building, and the materials used may be variable according to the costs and budgets or the ease of availability. In addition, most of the city’s religious buildings have undergone restoration operations, and it is therefore more difficult in these cases to identify unrestored surfaces, unlike private buildings.

This work presents, for the first time, a historically accurate reconstruction of the materials and conservation methods used on the facades of historical buildings in Lecce (Apulia, Italy). Ten buildings of the city of Lecce, both civil and religious, have been selected in order to analyze the traditions widespread in the past in the field of buildings’ conservation in the Lecce area. Thanks to non-invasive or micro-destructive techniques (optical microscopy, micro-FTIR spectroscopy, pyrolysis–gas chromatography–mass spectrometry), the molecular marker characteristics of the materials and the products of degradation have been identified, deepening the knowledge of the mechanisms of degradation and interaction between the stone material, the conservation treatment and the surrounding environment. The research is configured as a tool for the knowledge of ancient traditions, of the local historical memory and of the degradation mechanisms of water-repellent finishes applied in the past. The work is therefore a valuable contribution to the planning of appropriate restoration strategies for stone monuments.

## 2. Materials and Methods

### 2.1. Samples

**Figure 1:**
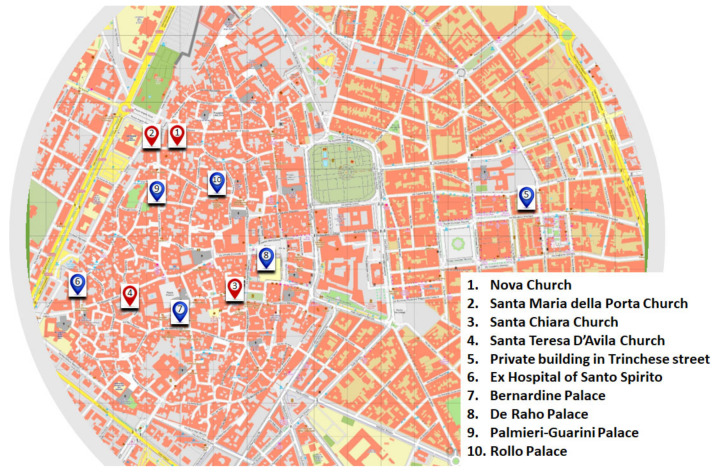
Map of the historical center of the city of Lecce (Apulia, Italy) and location of the historical buildings studied in the paper.

**Figure 2:**
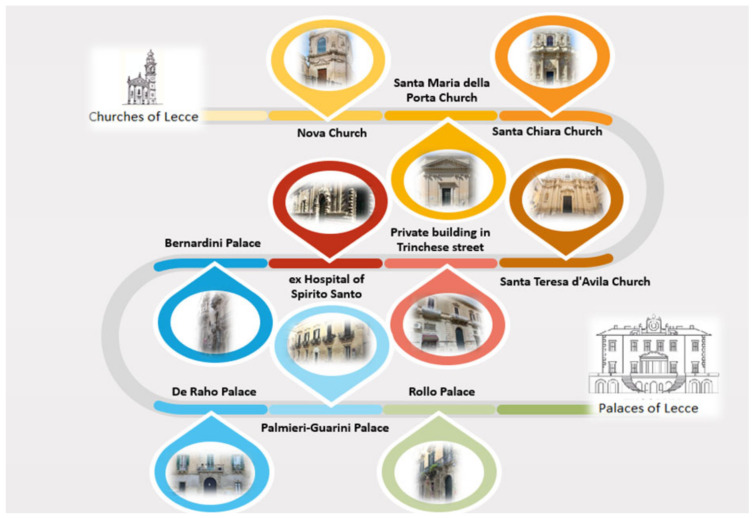
Synthetic representation of the investigated religious and civil buildings and main facades.

Ten buildings of historical-artistic value located in the city of Lecce (Apulia, Italy) were studied (Figure 1 and Figure 2, Figures S1 and S2), and more than 50 samples were collected from their facades (the most significant are shown in Table 1 and Table 2 and Figure S3). The identification of the samples was carried out taking into account the two main objectives of the work, namely the characterization of finishing techniques used in the past on stone facades and the study of degradation processes. Both civil and religious buildings of notable artistic value were selected in order to highlight possible differences in the methodologies of conservation of stone surfaces and investigate the surfaces not subjected to certified and published restoration interventions at the time of the research. The choice of the buildings to be investigated and of the sampling points was preceded by a first phase of knowledge of the artistic and historical context of the city of Lecce, carried out not only through archival and bibliographic research but also through visual analysis of the patina and coloring covering the facades of some buildings. From the visual analysis, the presence of “scialbature”, plastering and paintings is evident. In most cases, on the buildings of the historic center of Lecce, the presence of patinas of similar colors has emerged: the surfaces appear covered with beige, yellowish, orange, brown and especially pink patinas. Evidence of deterioration and alteration is also present on the stone surfaces, such as black crusts, saline efflorescence or subflorescence, alveolization, exfoliation, capillary rise of water and sulfation [19]. Overall, all the monuments investigated were built in “pietra leccese”, while some have a basement in “carparo”.

The sampling mainly concerned patinas and films on the facades of buildings. The buildings are subjected to protection restrictions (both the churches of the Archdiocese of Lecce and the buildings of the Associazione Dimore Storiche di Lecce (ADI)), so the sampling operations were guided by the officials of the Soprintendenza, who often indicated the areas to be sampled. Furthermore, in order to obtain representative scientific results that can be accredited, the sampling was extensive, and it was carried out at different points of the examined surfaces, through the use of gloves, tweezers and sterile containers. For each selected area of the surface, several samples were taken, ranging in size from a few millimeters to fragments of a few centimeters, both of the surfaces and in rare cases of the underlying stone. All sampling operations were documented (see Figures S1 and S2). For each analysis, at least five measurements were performed on each sample, in order to obtain scientifically meaningful results. A brief description of the monuments investigated is given in the following paragraphs (see Figure 1 and Figure 2). In Table 1 and Table 2, a synthetic description of the selected samples and their provenance are reported.

#### 2.1.1. Religious Buildings of Lecce (Apulia, Italy)

*Santa Maria della Porta Church or San Luigi Church*

The church was built in 1606 by expanding an ancient chapel of 1548 outside the walls of Porta Napoli, on Giuseppe Palmieri street of Lecce. The building was subsequently rebuilt between 1852 and 1858 in neoclassical style. The facade has an architraved portal flanked by high pilasters, with Ionic style capitals on which the architrave and the triangular pediment are located. The facade and the side walls show an uneven coloring; in the areas near the access stairs leading to the church, the stone surface is pale yellow, and there is a grey color in the degraded parts and in the most exposed areas of the projecting elements of the facade. In the upper part of the facade, instead, there is a slightly pinkish shade; this coloration becomes predominant in the area situated at about two meters of height from the walking surface, on the pilasters at the sides of the door of access to the church and in the area under the architrave. The entire building was restored in 1917. However, we do not know the technical details, and the materials used in the restoration operations are unknown.

*Nova Church or Church of the Natività della Vergine*

Nova Church is located on Idomeneo street in Lecce and was built between 1779 and 1782 on the area of an ancient 15th-century church (1470), thanks to the project of the Neapolitan engineer Carlo Salerni. The external facade was realized in Rococo style, prevalent at the end of the 18th century. In fact, it presents a polygonal development with two orders, divided by a notched cornice resting on four Corinthian pilasters, surmounted by pinnacles in the shape of flaming vases and connected by four festoons. The predominant color is the light yellow of Lecce stone, but some areas such as the columns decorating the sides of the portal, the pilaster of the upper floor (on the right of the central window) and several other areas of the lateral wall of the church have an intense yellow color. Static and conservative restoration interventions were attested in 2005. In 2009, operations of static consolidation and restoration from humidity were carried out; in 2013, operations of cleaning, protection and consolidation of the facade and the lateral surface were carried out through the use of an ethyl silicate as consolidating agent and a hydropolymethylsiloxanic polymer as protective.

*Santa Chiara Church*

Santa Chiara Church is located in Vittorio Emanuele square, Lecce. It was built between 1429 and 1438 and renovated between 1687 and 1691. The front elevation is characterized by two superimposed orders: The lower order is made up of Corinthian-style pilasters, with the sumptuous main portal with the coat of arms of the Seraphic Order at the top, surrounded by empty side aedicules decorated only at the top with medallions. The upper order, instead, is divided into three parts: the central one houses the window, and the side ones house niches without statues but are equally decorated. With the exception of some areas of the projecting elements in which we can observe a grey color due to superficial deposits and to rainwater runoff, the main facade presents the light yellow color typical of Lecce stone. In some areas that develop especially around the decorative elements of the facade (niches, columns), on the upper floor of the main facade and along the side surface exposed on Via degli Ammirati, the color of the stone material becomes orange. Between 1995 and 1999, the restoration of the facade was carried out, which included: cleaning with the use of two biocidal substances (Metatin 4% and Velpar 2%); cleaning with a brush and micro-sanding with alumina; consolidation with Estel 1000; filling with slaked lime, Lafarge lime and pigmented stone powder; protection with Silirain 50; and localized application of pigmented lime.

*Santa Teresa d’Avila Church*

Santa Teresa d’Avila Church is located on Giuseppe Libertini street (Lecce). It was probably designed by Giuseppe Zimbalo and built starting from 1620, on the area occupied by two ancient chapels dedicated to San Nicola and Santa Venera. Its construction was completed in 1630.

The facade is divided into two orders: the lower order is characterized by columns and two niches that house the stone statues of St. John the Baptist and St. John the Evangelist; the upper order is characterized by a large window and floral decorations.

The main facade has an anomalous aspect compared to the other ecclesiastical buildings of the city of Lecce. The lower order is characterized, in fact, by a pinkish coloration which becomes darker until it assumes an orange shade along the interstices of the columns, on the other decorative elements (coat of arms, frame, etc.) and on the sides of the portal of access to the church; the upper order presents instead a yellowish coloration typical of the stone material used for the construction. The facade was restored in 2004. As consolidating material, an ethyl silicate was applied; as protective material, a hydropolymethylsiloxanic polymer. Before the restoration operations carried out in 2004, two fragments, named LCT1 and LCT4, were taken from the facade.

#### 2.1.2. Civil Buildings of Lecce (Apulia, Italy)

*Ex-Hospital of Spirito Santo*

The old hospital, with the annexed chapel of the Holy Spirit, is located on Giuseppe Libertini street, in the historical center of the city of Lecce. It was built in 1394 and rebuilt in 1548, according to the project of the military engineer Gian Giacomo dell’Acaja. A strongly projecting cornice divides the facade into two orders. The lower part is marked by pairs of pillars resting on a high rusticated basement, a work that returns in the frame of the three large windows and in the portal, while the upper part is characterized by windows with simple frames. Above the portal, there is a window with a round arch, and above this, there is a decorated round-headed frame. The facade has an off-center portal and the cornice is interrupted by two framed windows only on one side. The main facade has a yellowish coloration; in fact, there are visible residues of “scialbature” and signs of impregnation of organic nature that over time have darkened the stone material, especially within the cavities of the decorative elements (rusticated base and grooves of the pilasters). There are no known restoration interventions that have targeted the facade before the sampling operations. 

*Private building on Trinchese street n.18*

The private palace is located on Trinchese street n.18, in the historical center of Lecce. There is no certain information about the year of construction of the building. According to the literature inherent to the urban culture of the 19th-century Lecce, in which the road takes on great importance for the opening of new axes and for the regularization of existing ones, and according to the architectural features of the building, it is possible to place the realization in the 19th century. The facade is characterized by a simple volume and a symmetry of the elements. The ground floor is characterized by a central entrance with ashlar decoration, next to which are placed the openings of the stores. The second floor, on the other hand, is characterized by five balcony windows, one central to the portal and four lateral ones, delineated by gables and pilasters. The facade has a pinkish hue that becomes lighter near the side openings of the second floor and just below the terminal cornice. Signs of deposition of carbonaceous particles that have darkened the surface over time can be observed in the internal areas of the gable, under the balconies and on the supports. No restoration interventions on the facade have been documented, while the interior has been remodeled several times. During the internal renovation works, splendid floor mosaics and frescoes decorating the vaults came to light, probably dating back, according to historians, to between the end of the 19th century and the beginning of the 20th century.

*De Raho Palace*

De Raho Palace is located on Dasumno street n.18 in Lecce. The palace was built in the 16th century and renovated in the 18th century by Mauro Manieri and his son Emanuele, also creators of the scenographic entrance. The main facade is developed on the ground floor with the scenographic portal, embellished by the shell motif that stands out in the upper part of the same; the upper floor, architecturally simpler, is characterized by the central door with a balcony entrance and by two side openings also equipped with small balconies resting on simple shelves. The front elevation and the side walls present a non-homogeneous coloration; on the ground floor, the signs of successive tampering are visible in the whiter plastered areas, while the yellow color of the stone is visible for the rest of the surface. In the decorative elements of the portal, the color becomes dark yellow, almost red, while the lateral volutes also show grey patinas and blackening. In the upper part of the facade, however, the color changes to a slightly pinkish hue, which is most evident near the decorative elements of the windows and on the supports of the two side balconies. Up to the time of the sampling operations, no restoration work was known.

*Bernardini Palace or Andretta Palace*

Bernardini Palace, also called Andretta Palace, is located on Arcivescovo Petronelli street n.18 in Lecce. The palace was owned by Bishop Domenico Antonio Bernardini (1645–1723) at the end of the 17th century. Later, it passed to his nephew, who enlarged and modernized it by rebuilding the facades, the interiors, the staircase and the atrium. After his death in 1759, the reconstruction continued for some years thanks to his wife, but it was never completed. The property of Palazzo Bernardini passed to Mascoli became in 1836 and then to Angelo Andretta in 1892. Valerio, Angelo Andretta’s son, completed the Bernardini project with the elevation of the second floor and the construction of the building that forms the backdrop to the atrium, instead of the garden. The facade presents the ground floor and the second floor dominated by the central stone balcony and the entrance with side pilasters; small trilobated windows and windows with metal balconies frame the entrance portal. The front elevation presents a light color quite homogeneous, tending to light gray in the undercuts of the pilasters of the central entrance, near the central balcony, both in the underlying areas less exposed to the weather and in the projecting areas. On the first and second floors, the light color becomes slightly more yellowish along the frames of the French doors. No restoration work was documented at the time of sampling.

*Rollo Palace*

The palace is located on Vittorio Emanuele II street n.14, Lecce. The original structure of the palace dates back to the second half of the 16th century. A first restructuring with consequent enlargement of the building was carried out in the 18th century. The facade is divided into three levels. Under the windows of the second floor, there is a row of balconies supported by corbels, while the windows are limited by semicircular tympanums, which rest on pilasters, considerably protruding from the surface. On the ground floor, the portal is built by means of an arch on which the coat of arms of the Rollo family, ancient owners of the building, is evident. The facade has a yellowish coloration that becomes darker in the upper levels, especially in the decoration of the entrance portal, in the cavities of the noble coat of arms and under the brackets supporting the balconies of the second floor and the sides of the opening of the main balcony. A gray color is instead observed in the points subject to water runoff on the surface of the semicircular gables of the second floor and under the balconies, becoming black on the second floor in the areas affected by the phenomenon of capillary rise of water. No restoration work was documented at the time of sampling.

*Palmieri-Guarini Palace*

Palmieri-Guarini Palace is located on Giuseppe Palmieri street n.42, Lecce. We do not know the years of construction and the builder, but we suppose due to the architectural features that it is of the 16th century. Owned by the Franchini family in the 17th century, it then passed to the Palmieri family, marquises of Martigliano, who during the 18th century carried out extension works, and finally passed to the Guarini dukes. The building has two elevations: the oldest located on Giuseppe Palmieri street is characterized by seven windows on the main floor, including the central one in correspondence of the Catalan-durazzo portal, in turn flanked by six windows, while the second 18th-century elevation is located in Ignazio Falconieri plaza, made by Manieri through the creation of six window balconies and framed by plant and floral motifs (on the second floor) and the portal and four windows (on the ground floor). The main facade is delimited by two angular columns with the heraldic coat of arms. While the 18th-century facade does not present particular stone colorations, due to post-construction bleaching operations, the facade on Giuseppe Palmieri street, which has not been subjected to restoration operations, at least documented until sampling, presents traces of color. Reddish colors can be observed especially in the most hidden points, such as the frame of the portal and the window frames, while black crusts occupy the internal parts of the portal. The restoration interventions of the adjacent facade on Giuseppe Palmieri street are not known.

### 2.2. Analytical Methods

A multi-analytical approach was used to characterize the materials and their distribution, alteration and degradation products; to study the phenomena occurring at the material/environment interface; and to identify changes due to aging. The non-destructive or micro-destructive analytical techniques used are given in the following section.

#### 2.2.1. Optical Microscopy

A Nikon Eclipse 80i Optical Microscope (Nikon Instruments s.p.a., Firenze, Italy), equipped with a X-Cite 120 source and a high-sensitivity digital camera (Nikon Instruments s.p.a., Firenze, Italy) was used for sample observation. In addition, Nikon B-2A and UV-2A filters (Nikon Instruments s.p.a., Firenze, Italy) and magnification up to light were used [10].

#### 2.2.2. Attenuated Total Reflectance Infrared Spectroscopy (ATR-FTIR)

**Figure 3:**
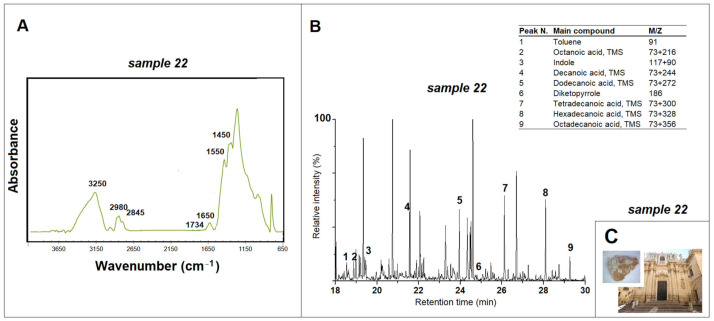
(**A**) ATR-FTIR spectrum for sample 22 taken from the *Santa Teresa D’Avila Church* (Lecce) and peaks related to the protein material; (**B**) pyrogram of sample 22 obtained by silylation (HMDS) and table with the main compounds identified; (**C**) images of *Santa Teresa D’Avila Church* and of sample 22.

**Figure 4:**
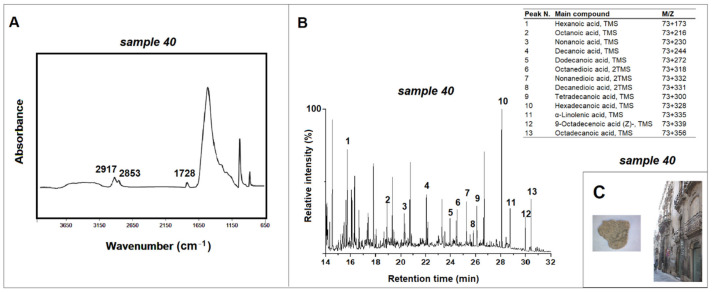
(**A**) ATR-FTIR spectrum for sample 40 taken from the *Bernardini Palace* (Lecce) and peaks related to the lipidic material; (**B**) pyrogram of sample 40 obtained by silylation (HMDS) and table with the main compounds identified; (**C**) images of *Bernardini Palace* and of sample 40.

**Figure 5:**
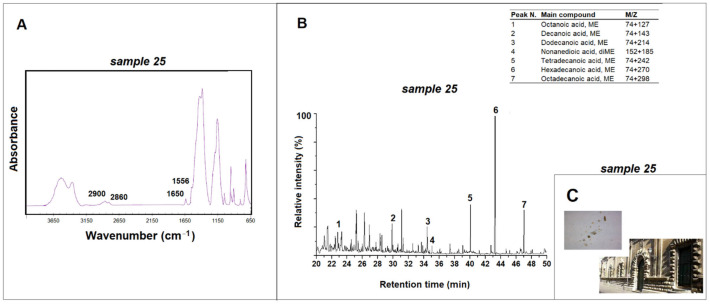
(**A**) ATR-FTIR spectrum for sample 25 taken from the *Ex-Hospital of Spirito Santo* (Lecce) and peaks related to the proteic material; (**B**) pyrogram of sample 25 obtained by methylation (TMAH) and table with the main compounds identified; (**C**) images of *Ex-Hospital of Spirito Santo* and of sample 25.

**Figure 6:**
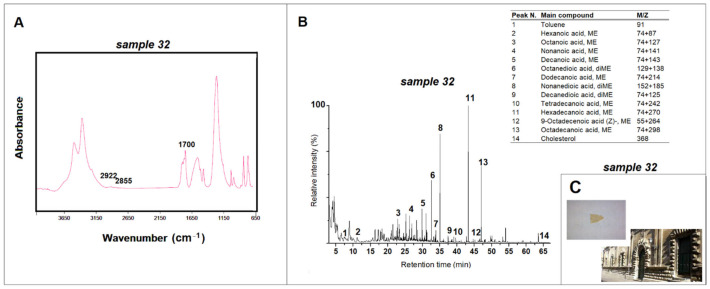
(**A**) ATR-FTIR spectrum for sample 32 taken from the *Ex-Hospital of Spirito Santo* (Lecce) and peaks related to the lipidic material; (**B**) pyrogram of sample 32 obtained by methylation (TMAH) and table with the main compounds identified; (**C**) images of *Ex-Hospital of Spirito Santo* and of sample 32.

**Figure 7:**
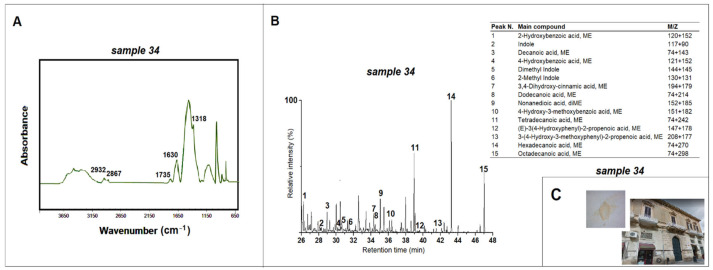
(**A**) ATR-FTIR spectrum for sample 34 taken from the *private building on Trinchese street n.18* (Lecce) and peaks related to the lipidic material and calcium oxalate; (**B**) pyrogram of sample 34 obtained by methylation (TMAH) and table with the main compounds identified; (**C**) images of the *private building on Trinchese street n.18* and of sample 34.

The infrared spectra were obtained using a Cary 600 Agilent Technologies FTIR spectrometer (Agilent Technologies, Milano, Italy). The spectrometer is connected to an optical microscope (Cary 610model, Agilent Technologies, Milano, Italy) equipped with a wide-band MCT detector and a ZnSe ATR crystal. The measurements were performed in attenuated total reflectance, and ATR-FTIR spectra were collected in the spectral region between 650 and 4000 cm^−1^, with a resolution of 4 cm^−1^ and averaging 64 scans. For each sample, five measurements were made and a standard deviation less than 5% has been calculated. All spectra were baseline corrected. The ATR-FTIR spectra shown in Figure 3, Figure 4, Figure 5, Figure 6 and Figure 7 correspond to the average curve obtained from five repeated analyses on the same indicated sample [10].

#### 2.2.3. Analytical Pyrolysis Coupled to Gas Chromatography–Mass Spectrometry (Py-GC/MS)

The Py-GC/MS analyses were carried out on a Curie temperature pyrolyzer (Pilodist, Bonn, Germany) fitted on the injector of a 6890N GC (Agilent Technologies, Palo Alto, CA, USA) connected to a 5973inert Mass Selective Detector (Agilent Technologies, Palo Alto, CA, USA). Samples (of about 0.1–0.2 mg) were loaded into a ferromagnetic cup, inserted into a quartz pyrolysis chamber and pyrolyzed at 670 °C for 10 s [10].

To increase the volatility of components, samples were subjected to thermally assisted hydrolysis–methylation pyrolysis according to a procedure already described [18,20] or to silylation [3]. In the first case, samples (less than 1 mg) were mechanically homogenized with 5 μL of tetramethylammonium hydroxide (TMAH) (2.5% in MeOH) to assist the hydrolysis and methylation of compounds and, after solvent evaporation at 60 °C for 30–35 min, placed in ferromagnetic tubes closed at the lower end having the selected Curie point temperature. Operating conditions have been reported in previous work [18]. Derivatization with hexamethyldisilazane (HMDS, Sigma-Aldrich, Milan, Italy) was carried out by adding 5 μL of neat HMDS to samples before loading in the ferromagnetic cup. The pyrolyzer interface was kept at 200 °C; injector and GC/MS interface were maintained at 300 °C. Pyrolysates were injected in splitless mode and separated on a Restek RXI 5-Sil MS fused silica capillary column (Crossbond 1,4-bis(dimethylsiloxy)phenylene dimethyl polysiloxane, 30 m × 0.25 mm i.d. × 0.25 mm film thickness with 10 m long Integra Guard pre-column, Restek Corporation, Bellefonte, PA, USA) using helium at 1.0 mL/min as carrier gas and the following temperature program: 40 °C isothermal for 10 min, 5 °C/min up to 300 °C and isothermal for 5 min [10,18].

The data were analyzed using the MS Chemstation software (Agilent Technologies, Palo Alto, CA, USA). The values and percentages calculated from the Py-GC/MS analyses refer to the average values of the five measurements of each sample examined (standard deviation less than 5%). The identification of the compounds was carried out by computer matching of the resulting mass spectra with the NIST mass spectral library or by comparison with mass spectra of known standards [10,18].

## 3. Results and Discussion

Optical microscope observation was performed directly on powdered samples or on fragments that had not undergone any preparation, so that during chemical analysis it was possible to operate directly on more external and representative areas of the samples. The analysis provided surface morphological information of the samples and highlighted the presence in some cases of organic material, through the observation of UV fluorescence; the use of a high-resolution camera connected to the instrument also allowed obtaining photographic documentation of what was observed under the optical microscope. Moreover, optical microscopy also provided information on the homogeneity/inhomogeneity, thickness and continuity/discontinuity of the film and patinas [21]. The ATR-FTIR analysis, also carried out directly on the samples as they were, thanks to the use of the mode of analysis in total attenuated reflection, led to the identification of functional groups of molecules present in the samples through the determination of the respective absorption bands of infrared radiation in the spectra obtained. Qualitative and semiquantitative information was obtained on the inorganic compounds (related to the stone support and to the presence of degradation compounds) and organic compounds (related to surface finishing techniques or to the presence of pollutants) present in the samples. Py-GC/MS analyses were performed on both samples not subjected to any pretreatment and derivatized samples. Both methods were used to obtain as much information as possible on the samples studied through the comparison of the results obtained with the data in the literature: Today, thermally assisted methylation remains the most widely used method on a wide range of organic materials, including those used in the field of cultural heritage. Py-GC/MS analyses have led to the identification of specific markers of organic compounds, allowing the characterization of pollutants and surface finish treatments present in the studied samples. Overall, the non-destructive and micro-destructive analyses showed the presence of different ancient finishing methodologies used on the Lecce stone and some products of degradation. The most significant results obtained for the historic buildings considered in this study are described below. Only the analytical data for the most representative samples are reported in the following discussion.

### 3.1. Results of Religious Buildings of Lecce (Apulia, Italy)

Most of the samples taken from the facades of religious buildings located in the city of Lecce did not show the presence of ancient conservation treatments. The results depend mainly on the restoration operations probably carried out in some places on the surfaces of the churches, in addition to the natural process of degradation of the materials. The most significant data have emerged for the *Santa Teresa D’Avila Church*. Specifically, samples 22 and 23 taken on either side of the church portal prior to restoration operations in 2004 showed evidence of protein material (Figure 3). The ATR-FTIR spectra of these samples showed the presence of the specific absorption bands located at about 3400 cm^−1^, related to the stretching of the N-H bond, and around 1650 cm^−1^ and 1550 cm^−1^, attributed to the presence of amide I and amide II, as well as the peak located at 1450 cm^−1^ related to amide III, only in sample 22 [10,22,23]. There are also characteristic bands of methyl groups at approximately 2950 cm^−1^ and 2850 cm^−1^ and carbonyl groups at 1734 cm^−1^ attributable to the presence of lipid material [10,22]. Characteristic markers of milk appear in the respective pyrograms, such as toluene and indols (derived from tryptophan), pyrrole and derivatives (derived from hydroxyproline and proline, in quantities less than 50%) and compounds derived from the fragmentation of polysaccharides and fatty acids (FAs) [24,25,26]. Among these, some short- and medium-chain fatty acids are present in the pyrograms of the samples, such as C8:0, C10:0, C12:0 and C14:0, in addition to palmitic (C16:0) and stearic acids (C18:0). Their presence, the low percentage of dicarboxylic acids (ΣD%, only traces), the very low ratio of palmitic and stearic acids C16:0/C18:0 (equal to 3.32 and 2.83 in the samples 22 and 23, respectively), and the ratio between lauric and capric acids C12:0/C10:0 equal to 0.55 in sample 22 and to 0.35 in sample 23 confirm that milk was applied on the facade of the church [10,25,26,27].

The Py-GC/MS data, while confirming the presence of milk, previously suggested by ATR-FTIR analysis, do not, however, allow for the identification of the type. Historical sources report, for example, the use of milk diluted with water or mixed with talc [3,10]. In other cases, the use of goat, sheep or cow’s milk is specified [2,3]. The scientific literature generically shows in some cases the use of protein materials as binders in protective and aesthetic treatments on the surface of the stone [10,15,18,28]. In more specific cases, the use of sheep or cow’s milk has been better hypothesized; the origin of milk can sometimes be identified by the content of short- and medium-chain fatty acids and the ratio of lauric acid to capric acid (C12:0/C10:0) [29,30]. The value of the C12:0/C10:0 ratio of 0.35 measured in sample 22 [26,27] and the presence in the infrared spectrum of the peak still identifiable at 1734 cm^−1^ [10] could be associated with the use of sheep’s milk. However, the data are not conclusive, due to the presence of other pyrolyzed materials that may alter the value of the determined ratio.

Lower amounts of lipidic material were found in sample 1, collected from *Santa Maria della Porta Church*, and sample 7, collected from *Nova Church*, whose concentrations cannot be attributed with certainty to ancient stone finishing treatments.

In the study of the mechanisms of degradation, the ATR-FTIR analysis showed the presence of deterioration products, such as sulfates and calcium oxalates (Table 3), on the stone surfaces in almost all samples. Sulfates are present in considerable quantities in the forms of calcium sulfate monohydrate CaSO_4_·H_2_O and calcium sulfate dihydrate CaSO_4_·2H_2_O (gypsum): the former shows two infrared radiation absorption bands at about 3615 cm^−1^ and 3465 cm^−1^ and a peak at 1630 cm^−1^, respectively related to symmetric and asymmetric stretching and bending vibrations of the O-H bond [18]; on the other hand, gypsum shows absorption peaks characteristic of the hydroxyl group at 3555 cm^−1^ and 1680 cm^−1^ [22]. Regardless of the state of hydration, sulfates also show absorption bands characteristic of the stretching and bending vibration of the S-O bond at about 1122 cm^−1^ and 602 cm^−1^, respectively. Their presence in the analyzed samples can be attributed to sulfation phenomena, due to the attack of calcium carbonate of calcarenites by sulfuric acid: a very common deterioration process in urban environments [22].

Samples 3–4, 10–15 and 16–23 present calcium oxalates (Table 3), also very common deterioration compounds on stone surfaces, whose characteristic infrared bands are located at about 1620 cm^−1^, 1320 cm^−1^ and 777 cm^−1^ [15,22]. Although it is difficult to discriminate by infrared spectroscopy between the usually more widespread monohydrate form of calcium oxalate, named whewellite Ca(C_2_O_4_)·(H_2_O), and the bihydrate form, named weddellite Ca(C_2_O_4_)·2(H_2_O), the determination in all samples of a single band related to O-H bonding at about 3400 cm^−1^, rather than multiple absorption peaks at 3470, 3336 and 3057 cm^−1^, would seem to indicate the presence of weddellite [21,31]. The causes of the formation of the calcium oxalate film have not yet been completely clarified: the most widespread hypotheses link their origin to the natural aging of the stone caused by chemical–physical reactions between the surface of the material and the environment, to biological activity or to ancient protective treatments [15,21,31,32]. However, their determination in samples in which the presence of ancient protective treatments has been attested supports the hypothesis of artificial origin: this is the case of samples 22 and 23 from *Santa Teresa D’Avila Church* in which milk-based protective treatments have been identified [15,31,32,33]. Different is the case of samples 16–21 taken from the same church facade, samples 3 and 4 taken from *San Luigi Church* and samples 9–15 taken from *Santa Chiara Church*, whose origin remains uncertain [15,21].

The results of the Py-GC/MS analysis led to the qualitative identification of organic pollutants such as aliphatic hydrocarbons (AHs), polycyclic aromatic hydrocarbons (PAHs) and fatty acid derivatization products (Table 3). Specifically, alkenes were determined in the pyrograms of samples 9, 19 and 21; naphthalene in samples 9 and 19; styrene exclusively in sample 19; toluene in sample 21; and fatty acids in samples 1, 7 and 9. No notable differences were found between the samples taken from the facades of architectural properties subjected to restoration operations and those not subjected to recent conservation interventions. From the analytical results, the possible sources of pollution have been identified: most of the organic compounds identified through Py-GC/MS analysis in the samples taken from the urban area are attributable to anthropogenic sources (vehicular traffic, incomplete combustion of oil, tar, bitumen, oil, etc.) [34,35,36]; the presence in the pyrograms of fatty acid methyl esters could instead be attributable to forms of microbiological degradation [34,35,36], whose presence has not, however, been confirmed by observation of the samples under an optical microscope or to the deterioration of ancient finishing treatments.

### 3.2. Results of Civil Buildings of Lecce (Apulia, Italy)

Regarding the study of ancient finishing techniques used on civil buildings in the Lecce city, important results have been obtained.

The samples that have provided the most significant data are: sample 40 taken from *Bernardini Palace*; sample 38 sampled from *De Raho Palace*; sample 41 sampled from *Rollo Palace*; samples 25, 27, 28 and 32 sampled from *Ex-Hospital of Spirito Santo*; and sample 34 sampled from the *private building on Trinchese street n.18*.

In sample 40, the ATR-FTIR analysis showed the presence of absorption bands of infrared radiation typical of fatty acids, such as characteristic peaks at 2917 cm^−1^ and 2853 cm^−1^ related to the C-H stretching and at 1728 cm^−1^ related to the C=O stretching (Figure 4) [10,22]. The presence of lipidic material has been confirmed by Py-GC/MS analysis, which showed significant amounts of dicarboxylic acids (DCAs), such as azelaic (C9) and suberic (C8) acids, the main stable products found in aged pictorial films of the autoxidative processes involving the unsaturated acyl chains of siccative oils, during drying and aging [25,26]. In addition, significant amounts of saturated and unsaturated fatty acids are present in the pyrograms of sample 40. The presence of all these markers, 65.38% monocarboxylic acids, 21.49% dicarboxylic acids, the azelaic and palmitic acids ratio of 0.05, and the oleic and stearic acids ratio of 0.51 confirm the use of a siccative oil in the original finishing layer of sample 40 [10,25,26]. In fact, the drying oils are mixtures of triglycerides of saturated and unsaturated fatty acids, and this drying oil in particular would be walnut oil, as can be deduced from the measurement of the ratio of palmitic and stearic acids equal to 2.09 [3,10,27].

The ATR-FTIR spectra of samples 38 (taken from *De Raho Palace*) and 41 (collected from *Rollo Palace*) showed the presence of protein materials. Sample 38 showed specific absorption bands located at 3870 cm^−1^, related to the N-H stretching; at 1640 cm^−1^ and 1539 cm^−1^, attributed to the presence of amide I and II; at 1397 cm^−1^ related to amide III [10,22,37]. In the ATR-FTIR spectrum of sample 41, there are infrared peaks located at about 3400 cm^−1^ (N-H stretching), 1680 cm^−1^ and 1543 cm^−1^ (amides I and II), and 1409 cm^−1^ (amide III). The characteristic bands of methyl groups (at about 2950 cm^−1^ and 2850 cm^−1^) and carbonyl groups (at about 1730 cm^−1^) are also present in both infrared spectra [10,22].

The results of the Py-GC/MS analysis confirm the use of milk as a protective material used for the Lecce stone [10,26]. In the respective pyrograms, in fact, markers characteristic of milk appear, such as toluene and indole, which derive from tryptophan, and pyrrole and its derivatives, which derive from proline [10,20]; the latter are present in traces in the pyrogram of sample 38. In both pyrograms there are also some compounds derived from the fragmentation of polysaccharides and fatty acids: in addition to palmitic and stearic acids, short-chain (in sample 38) and medium-chain C6, C8, C10, C12 and C14 (in samples 38 and 41) are evident. The low percentage of dicarboxylic acids and the C16:0/C18:0 and C12:0/C10:0 ratios confirm that milk was applied on the facade of the buildings [10,25,26,37].

The ATR-FTIR spectra of samples 25, 27 and 28 taken from the *Ex-Hospital of Spirito Santo* showed the presence of absorption peaks related to methyl groups and located at about 2900 cm^−1^ and 2860 cm^−1^ (Figure 5). The infrared spectrum of sample 25 also shows amide bands at 1650 cm^−1^ and 1556 cm^−1^, indicating the presence of proteinaceous material (Figure 5) [26,37]. The results of Py-GC/MS analysis indicate the presence of saturated and unsaturated fatty acids in these samples. Azelaic acid (C9) is also present in low concentrations in samples 25 and 27. From the percentages of monocarboxylic acids (between 30 and 40%) and dicarboxylic acids (less than 10%) and the ratio of azelaic and palmitic acids (less than 0.1%), the presence of proteinaceous material in the samples analyzed can be deduced [26]. However, the absence of toluene, indole, pyrrole and pyrrole derivatives leads to the exclusion of the use of milk or animal glues as waterproofing agents, while the absence of cholesterol and hexadecanonitrile excludes the use of egg yolk [25,26]. An egg-white-based finishing treatment was therefore used, although its use on large surfaces seems to have been obsolete in the past according to literary sources and limited moreover to the gluing with sugar addition of stone parts, rather than to the realization of finishes for conservation purposes [3].

Sample 32 taken from the same building deserves separate consideration (Figure 6). The ATR-FTIR spectrum shows the peaks associated with the presence of lipidic material, located at 2922 cm^−1^ and 2855 cm^−1^, related to the C-H methyl groups, and a peak at 1700 cm^−1^, characteristic of the C = O carboxylic functional group [10,22,26]. Further information comes from the qualitative and semiquantitative analysis carried out using Py-GC/MS that shows the probable use of a mixed technique. In this case, the use of an emulsion of egg and siccative oil is indicated by the presence of cholesterol (and its degradation products), traces of toluene and monocarboxylic (39.52%) and dicarboxylic (equal to 25.04% and therefore higher than 10%, as reported in the literature) acids in the pyrogram, as well as by the azelaic and palmitic acids ratio of 0.34 [24,26].

Sample 34, taken from a *private building on Trinchese street n.18* (Lecce), is of particular interest compared to the other samples analyzed. The ATR-FTIR spectrum (Figure 7) shows absorption bands of infrared radiation at 2932 cm^−1^ and 2867 cm^−1^ related to C-H bond stretching. Moreover, absorption bands at 1630 cm^−1^ and 1318 cm^−1^ related to calcium oxalates are present [10]; calcium oxalates are usually found on surfaces affected by artificial finishes [15], and such absorption peaks remain together with methyl groups specifically after the aging of surface treatments based on some plant species, according to a previous experimental study [10]. Py-GC/MS analyses show predominantly the presence of indoles, lignin degradation compounds, polysaccharides and fatty acids [24]. The presence of methyl groups and significant amounts of disaccharides and the absence of toluene and pyrroles (characteristic markers of milk together with indoles and thermal degradation products of polysaccharides) confirm the use of a surface finishing technique based on polysaccharide material, already suggested by FTIR analysis [18]. The pyrogram of sample 34 (Figure 7) also shows the presence of some phenolic acids and flavonoids, characteristic markers of *Opuntia Ficus-Indica* leaf, confirming the use of this species for stone surface protection [18,38,39]. *Opuntia Ficus-Indica* is a plant belonging to the Cactaceae family and is very widespread in Apulia. Its leaves are rich in water, carbohydrates, waxes, phenolic compounds, minerals, oxalates and fatty acids. These substances have now led to its widespread use in various application fields due to its antimicrobial, adhesive and water-repellent properties. Even if the leaf of *Opuntia Ficus-Indica* is an unusual material, its use for the conservation of surfaces is not surprising today, since it has already emerged in a previous study on the facade of the *Church of San Giorgio* (Melpignano, Lecce) [18].

ATR-FTIR analyses (Table 3) showed the presence of sulfates, derived from the sulfation process of calcarenites by sulfuric acid, a phenomenon that affected all the civil buildings studied [22]. Calcium oxalates are also present, especially in the weddellite form Ca(C_2_O_4_)·2(H_2_O) [21,31], whose origins seem to be different: on the surfaces where protective treatments based on proteinaceous material (e.g., milk or egg on *Ex-Hospital of Spirito Santo* and the *De Raho* and *Rollo Palaces*), lipidic material (oil alone or together with egg on *Ex-Hospital of Spirito Santo* and *Bernardini Palace*) or polysaccharidic material (*private building on Trinchese street*), the causes may be anthropogenic, as supported by the scientific literature [10,15,33]. Some doubts about the origin of the calcium oxalates remain instead for the samples in which fatty acids were found by Py-GC/MS analysis, but in quantities limited enough to be attributed to superficial finishes applied voluntarily for aesthetic and/or conservation purposes. Few samples (43, 29, 31) present nitrates (Table 3), identified by the characteristic absorption peaks present in the ATR-FTIR spectrum at 1371 cm^−1^ and 1398 cm^−1^ [22]. These compounds are usually derived from the deposition of atmospheric particulate matter or from the process of corrosion of calcite by gaseous nitrogen oxides, present in the polluted urban atmosphere [34,35,36]. Additional sources include also biological activities or bird droppings [21]. The results of the Py-GC/MS analysis (Table 3) led to the qualitative identification of organic pollutants such as aliphatic hydrocarbons (AHs), polycyclic aromatic hydrocarbons (PAHs) and fatty acid derivatization products; in particular, the latter are those most commonly found in civil buildings, such as in the samples taken from the *Ex-Hospital of Spirito Santo* and *Palmieri-Guarini Palace*. Their origin may be associated with forms of biological degradation or anthropic causes [21,35].

## 4. Conclusions

Throughout this work, a considerable amount of samples from buildings of historical interest in the city of Lecce (Apulia, Italy) have been studied through the use of non-invasive or micro-destructive analytical techniques. For the first time, an accurate reconstruction of the materials and ancient techniques used for the protection of “pietra leccese” was carried out. Furthermore, the use of protein, lipid and polysaccharide materials for the protection of “pietra leccese” surfaces was demonstrated. Among these, the study confirmed that proteinaceous protective treatments were the most commonly used, both on religious and civil buildings, although these materials are more easily susceptible to degradation over time compared to lipid materials. Evidence of the use of unconventional materials, such as the leaf of *Opuntia Ficus-Indica*, in the field of the restoration of cultural heritage has also been demonstrated. Finally, the study of degradation products showed that all buildings, even those recently restored, are subjected to degradation phenomena typical of carbonate matrix materials in the urban environment, mainly due to anthropogenic causes. Future research will be focused on the porosity of the stone substrate in order to further investigate the phenomena of degradation and better understand the causes that led to the preservation of protective treatments in some areas rather than others. It would also be interesting to create a database of materials and techniques used in the past, which could be useful to superintendents and restorers who have to intervene for conservation purposes on buildings.

## Figures and Tables

**Table 1:** Description and provenance of the samples taken from religious buildings. All samples show patinas, films or efflorescence, according to UNI 11182:2006 classification.

Monument		Sample	Provenance	Description
***Religious building***	*Santa Maria della Porta Church (also known as San Luigi Church)*	1	left side of portal, inner part	yellow powder
		2	left side of portal, bottom part	yellow powder
		3	left side of portal, external part	pink powder
		4	right side of portal, external part	dark powder
	*Nova Church (also known as Church of the Natività della Vergine)*	5	side facade	red fragment
		6	side facade	white powder
		7	side facade	effluorescence
		8	side facade	pink powder
	*Santa Chiara Church*	9	right column of the portal, bottom part	dark fragment
		10	right side of the portal	grey powder
		11	right column of the portal, basement	yellow fragment
		12	right column of the portal, basement	grey fragment
		13	left side of portal, decoration	yellow powder
		14	left side of portal, decoration	grey powder
		15	left side of portal, decoration	yellow powder
	*Santa Teresa D’Avila Church*	16	left column of the portal, basement	yellow fragment
		17	left column of the portal	dark powder
		18	left column of the portal, basement cavity	dark powder
		19	left wall of the portal	yellow powder
		20	space between the first and second column on the right of the portal	dark powder
		21	right jamb of the portal	yellow powder
		22	main facade	yellow fragment
		23	main facade	yellow fragment

**Table 2:** Description and provenance of the samples taken from civil buildings. All samples show patinas or films, according to UNI 11182:2006 classification.

Monument		Sample	Provenance	Description
***Civil buildings***	*Ex-Hospital of Spirito Santo*	24	patina between the first pilasters on the left of the entrance	grey fragment
		25	patina between the first pilasters on the left of the entrance	grey powder
		26	pilaster to the left of the entrance	dark fragment
		27	pilaster to the left of the entrance	dark powder
		28	right jamb of the entrance	dark fragment
		29	left jamb of the entrance	grey fragment
		30	left jamb of the entrance	yellow powder
		31	second framed window on the left of the portal	orange fragment
		32	second framed window on the left of the portal	yellow fragment
	*Private building in Trinchese street*	33	base of the left parasta of the second window, elevated floor	yellow powder
		34	right parasta of the second window, elevated floor	yellow powder
		35	surface between the first and second window, elevated floor	yellow powder
		36	left parasta of the second window, elevated floor	yellow powder
	*De Raho Palace*	37	decoration on the left of the portal	red fragment
		38	portal frame, right side	yellow fragment
	*Bernardini Palace (also named Andretta Palace)*	39	window of the main balcony, elevated floor	yellow powder
		40	main balcony, elevated floor	grey fragment
	*Rollo Palace*	41	main balcony, elevated floor	yellow fragment
	*Palmieri-Guarini Palace*	42	left side of the entrance	red fragment
		43	entrance, inner part	dark fragment

**Table 3:** Major degradation compounds identified by ATR-FTIR spectroscopy and PY-GC/MS.

**Monument**		**Sample**	**ATR-FTIR Spectroscopy**	PY-GC/MS
***Religious building***	*Santa Maria della Porta Church (also known as San Luigi Church)*	1	Sulfates	Traces of FA
		2	Sulfates	/
		3	Calcium oxalates	/
		4	Sulfates, Calcium oxalates	/
	*Nova Church (also known as Church of the Natività della Vergine)*	5	Sulfates	/
		6	Sulfates	/
		7	Sulfates	Traces of FA
		8	Sulfates	/
	*Santa Chiara Church*	9	Sulfates	AHs, PAHs, Traces of FA
		10	Sulfates, Calcium oxalates	/
		11	Sulfates, Calcium oxalates	/
		12	Sulfates, Calcium oxalates	/
		13	Sulfates, Calcium oxalates	/
		14	Sulfates, Calcium oxalates	/
		15	Sulfates, Calcium oxalates	/
	*Santa Teresa D’Avila Church*	16	Calcium oxalates	/
		17	Sulfates, Calcium oxalates	/
		18	Calcium oxalates	/
		19	Sulfates, Calcium oxalates	AHs, PAHs
		20	Sulfates, Calcium oxalates	/
		21	Sulfates, Calcium oxalates	AHs, PAHs
		22	Sulfates, Calcium oxalates	/
		23	Sulfates, Calcium oxalates	/
***Civil buildings***	*Ex-Hospital of Spirito Santo*	24	Sulfates, Calcium oxalates	AHs, PAHs, Traces of FA
		25	Calcium oxalates	/
		26	Calcium oxalates	/
		27	Calcium oxalates	/
		28	Sulfates, Calcium oxalates	/
		29	Sulfates, Calcium oxalates, Nitrates	Traces of FA
		30	Sulfates, Calcium oxalates	Traces of FA
		31	Sulfates, Calcium oxalates, Nitrates	/
		32	Sulfates, Calcium oxalates	/
	*Private building in Trinchese street*	33	Sulfates	/
		34	Sulfates, Calcium oxalates	/
		35	Sulfates, Calcium oxalates	/
		36	Sulfates, Calcium oxalates	/
	*De Raho Palace*	37	Sulfates, Calcium oxalates	/
		38	/	/
	*Bernardini Palace (also named Andretta Palace)*	39	Sulfates, Calcium oxalates	/
		40	Calcium oxalates	/
	*Rollo Palace*	41	Sulfates, Calcium oxalates	/
	*Palmieri-Guarini Palace*	42	Sulfates, Calcium oxalates	/
		43	Calcium oxalates, Nitrates	AHs, PAHs, Traces of FA

## Data Availability

Not applicable.

## Supplementary Materials

The following supporting information can be downloaded at: https://www.mdpi.com/article/10.3390/ma15103658/s1, Figure S1: Religious buildings investigated and sampling points in red. (A) Santa Maria della Porta Church; (B) Santa Teresa D’Avila Church; (C) Nova Church; (D) Santa Chiara Church. Figure S2: Civil buildings investigated and sampling points in red. (E) Private building in Trinchese street; (F) Ex Hospital of Santo Spirito; (G) Rollo Palace; (H) De Raho Palace; (I) Palmieri-Guarini Palace; (L) Bernardine Palace. Figure S3: Photographs of the some representative samples: (A) sample 5, (B) sample 19, (C) sample 22, (D) sample 23, (E) sample 25, (F) sample 32, (G) sample 34, (H) sample 38, (I) sample 40, (L) sample 41.
