# Supplementary material for: Historically Accurate Reconstruction of the Materials and Conservation Technologies Used on the Facades of the Artistic Buildings in Lecce (Apulia, Italy)
Source: Materials (Basel). 2022 May 20;15(10):3658. doi: 10.3390/ma15103658 (PMC9145597; doi:10.3390/ma15103658)
Supplement: Supplementary file 1 [file materials-15-03658-s001.zip › materials-1736018-supplementary.pdf]

Supplementary materials

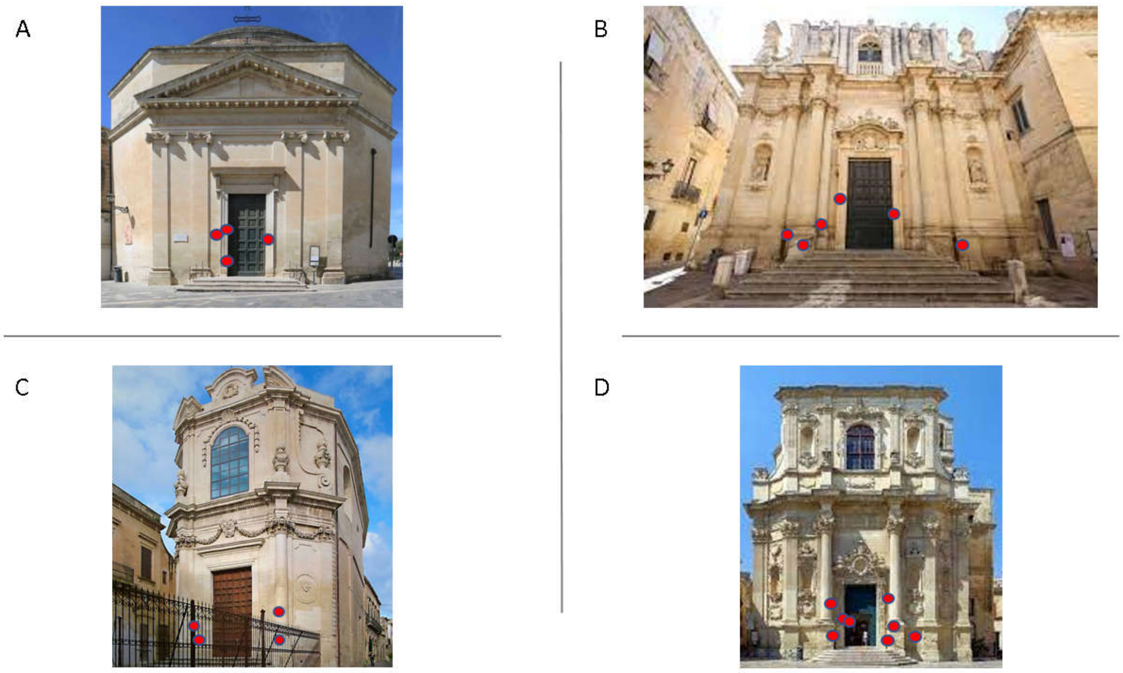

Figure S1. Religious buildings investigated and sampling points in red. (A) Santa Maria della Porta Church; (B) Santa Teresa D'Avila Church; (C) Nova Church; (D) Santa Chiara Church.

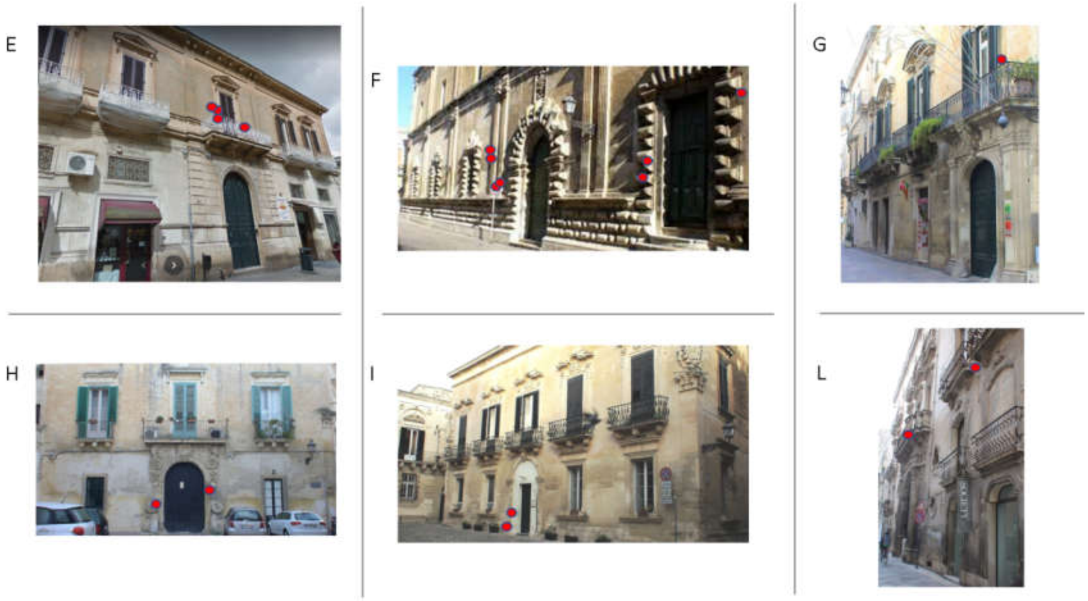

Figure S2. Civil buildings investigated and sampling points in red. (E) Private building in Trinchese street; (F) Ex Hospital of Santo Spirito; (G) Rollo Palace; (H) De Raho Palace; (I) Palmieri-Guarini Palace; (L) Bernardine Palace.

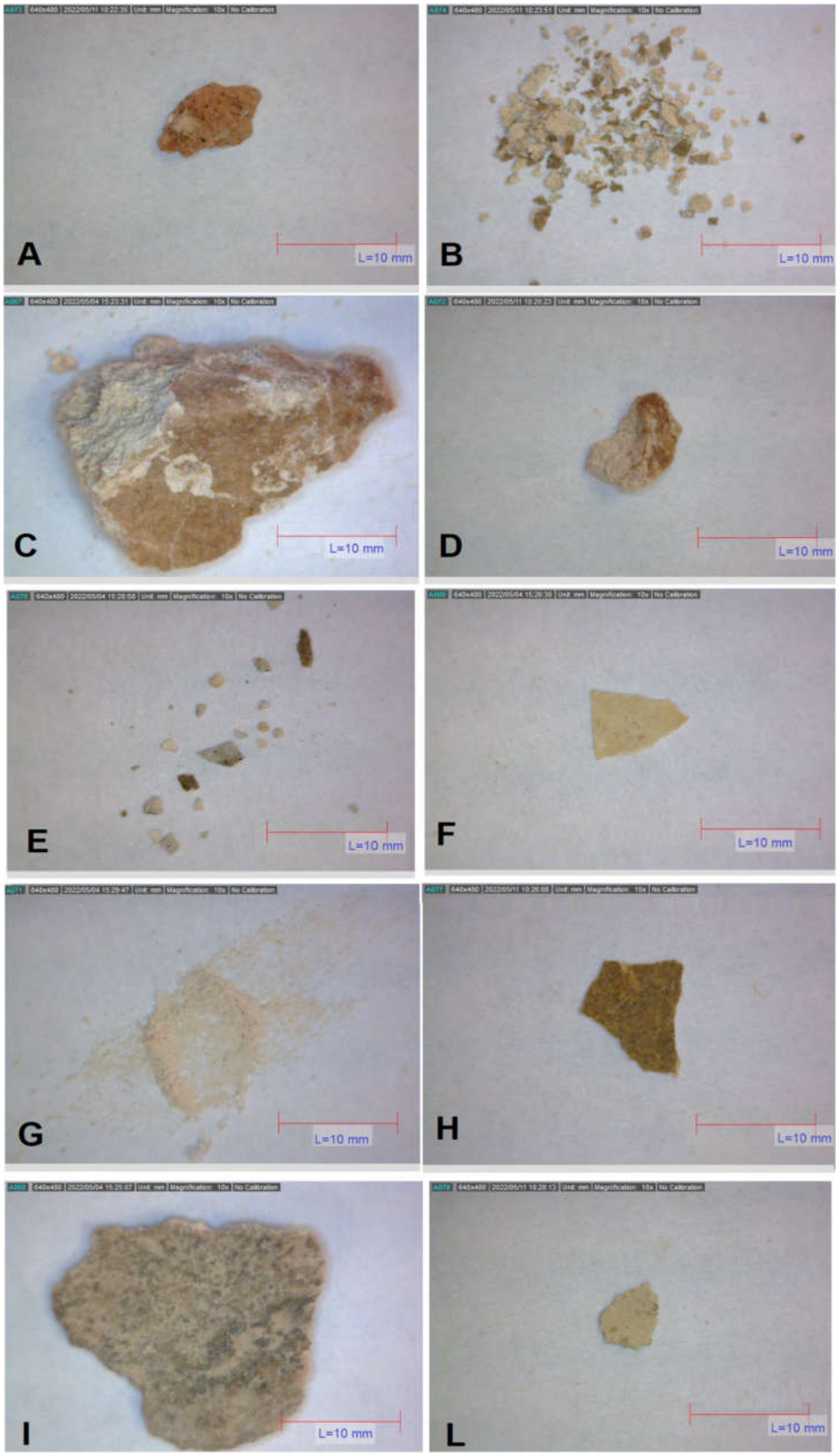

Figure S3. Photographs of the some representative samples: (A) sample 5, (B) sample 19, (C) sample 22, (D) sample 23, (E) sample 25, (F) sample 32, (G) sample 34, (H) sample 38, (I) sample 40, (L) sample 41.
